# An Association between Pancreatic and Cholestatic Biliary Disorders in Dogs

**DOI:** 10.3390/ani14050795

**Published:** 2024-03-04

**Authors:** Rebecca Dini, Eleonora Gori, Verena Habermaass, Ilaria Lippi, Simonetta Citi, Caterina Puccinelli, Veronica Marchetti

**Affiliations:** Department of Veterinary Sciences, Veterinary Teaching Hospital “Mario Modenato”, University of Pisa, Via Livornese Lato Monte, San Piero a Grado, 56122 Pisa, Italyeleonora.gori@unipi.it (E.G.); ilaria.lippi@unipi.it (I.L.); caterina.puccinelli@phd.unipi.it (C.P.);

**Keywords:** DGGR lipase, pancreatic injury, pancreatitis, biliary tree disease, cholestasis, chronic liver disease, hepatobiliary disease

## Abstract

**Simple Summary:**

The relation between biliary tree diseases and pancreatitis is not well-known in veterinary medicine. Anecdotally, hyperlipemia, pancreatic inflammation, and abnormalities of biliary flow are thought to play a role in the pathogenesis. The aim of the work was to study the frequency and features of pancreatic involvement in dogs with chronic biliary tree disease. We retrospectively included 81 dogs with both laboratory and ultrasound signs of chronic biliary tree disease. Medical records of the included dogs and their pancreatic involvement was recorded considering ultrasonographic features and/or biochemical alterations (Lipase DGGR). Fifty-eight dogs (72%) showed pancreatic involvement, suggesting that pancreatic status should be investigated in canine patients with chronic biliary tree disease.

**Abstract:**

Canine chronic biliary tree disease (CBTD) is a suspected risk factor for pancreatic injury. The aim of this study was to evaluate the frequency and features of pancreatic involvement in canine CBTD, and their relationship with hyperlipemia and its severity. CBTD was defined as the increase in at least two of ALP, GGT, total bilirubin, cholesterol, and a biliary tree abnormal abdominal ultrasound (graded mild to severe). Pancreatic ultrasound appearance was recorded and classified as acute/chronic. Dogs were divided into a PBD group (pancreatic and biliary disease) and BD group (only biliary tree disease). PBD group was subgraded into a “pancreatic injury” and “pancreatitis” group. Eighty-one dogs were retrospectively included: 56 in the PBD group and 25 in the BD group. Of the PBD group, 20 had pancreatitis (15 chronic and 5 dogs acute). US score was mild in 64 dogs and moderate in 17 dogs, and it was not associated with evidence of pancreopathy. Sixty-six dogs had hyperlipemia (mild = 27 dogs; moderate-to-severe = 39 dogs) and no association with pancreopathy was found. Pancreatic injury was more frequent than pancreatitis in CBTD dogs. Although both acute and chronic pancreatic injury may be present, chronic forms were more frequent. Pancreatic injury should be considered in CBTD patients due its possible clinical significance.

## 1. Introduction

Pancreatitis can be defined as an acute or chronic inflammatory disease that involves pancreatic and peri-pancreatic tissues, sometimes associated with multi-organ dysfunction [1,2,3]. In human medicine, one of the most common causes of acute pancreatitis (AP) is the presence of gallstones and subsequent gallbladder inflammation [4]. In veterinary medicine, gallstones are rarely found, and the relation between pancreatic and gallbladder abnormalities has been partially investigated [5]. Although the relation between biliary diseases and pancreatitis in veterinary medicine is not completely clear, hyperlipemia, abnormalities of the biliary flow, and pancreatic inflammation are thought to be implicated in the pathogenesis [6,7,8]. Dogs affected by chronic biliary tree disease (CBTD) and pancreatic inflammation may share similar clinical signs, due to mutual organ inflammation, hypothetically mediated by ascending bacterial colonization [9]. In a recent retrospective study by Peters et al., serum canine pancreatic lipase immunoreactivity (cPLI), and other hematological and biochemical parameters, together with biliary cytology and bacterial culture were evaluated in 56 dogs undergoing cholecystocentesis [10]. Serum cPLI was found to be significantly associated with bacterial cholecystitis, even though the pathogenic relation was not established [10].

Hyperlipemia is another common factor of pancreatitis and biliary tract diseases. Hyperlipemia, especially hypertriglyceridemia, is often recorded in patients with both chronic and acute pancreatitis [6]. Although patients with AP may have normal serum triglycerides, different profiles of lipoproteins have been highlighted between healthy dogs and dogs with AP [11,12]. Another study reported that hypertriglyceridemia may be the nexus between diabetes mellitus and the development of AP in dogs [13]. In a recent study, the prevalence of hypercholesterolemia in dogs with AP was higher than previously reported, and cholesterol concentration was strongly correlated with ALP and GGT activity, thus suggesting an association between cholestasis and hypercholesterolemia in dogs with AP [14].

On the other hand, biliary tree disorders are characterized by a mild increase in triglycerides and/or cholesterol [15]. Diffuse vacuolar hepatopathy and gallbladder mucocele are two hepatobiliary disorders associated with hypertriglyceridemia in dogs. Particularly, gallbladder mucocele has been reported in different endocrinopathies, such as hypothyroidism, diabetes mellitus, and hyperadrenocorticism, and it may be a common finding in breeds predisposed to primary hyperlipemia [16,17,18].

We hypothesized that pancreatic inflammation, both acute or chronic, may be a significant comorbidity in dogs with CBTD, and that hypercholesterolemia and/or hypertriglyceridemia may be associated with its development. The aim of this study was to evaluate the frequency and the features of pancreatic involvement in dogs with CBTD, and their relationship with the frequency and severity of hyperlipemia.

## 2. Materials and Methods

A retrospective cohort study was conducted on dogs with CBTD by searching in our veterinary hospital database (searching period January 2021–January 2023). CBTD was identified on the basis of ultrasonographic signs of biliary tract disease, and a concurrent increase in at least 2 of the following parameters: alkaline phosphatase (ALP) > 250 U/L, gamma-glutamyltransferase (GGT) > 1 1.0 U/L, total bilirubin (TB) > 0.30 mg/dL and cholesterol (CHOL) > 280 mg/dL [19] (SAT-450 biochemical analyzer; Assel S.r.l., Rome, Italy).

For each patient, a novel semi-objective ultrasound (US) score was used to grade the severity of the biliary tract disease. The score was based on ultrasonographic features of intra/extra hepatic biliary tracts and the gallbladder’s content and wall, evaluated according to the available literature [20,21,22,23,24,25]. The US parameters and their scoring system are reported in Table 1. A sub-score was given to every alteration and summed up. The total score expressed the gravity of the disease: mild (1–2), moderate (3–4), and severe (>5).

For each dog, serum DGGR lipase (U/L), cholesterol (CHOL, mg/dL), triglycerides (TRI, mg/dL), and data regarding pancreatic ultrasound were recorded. Serum DGGR lipase concentration was measured using a colorimetric method (SAT-450 biochemical analyzer; Assel S.r.l., Rome, Italy). Serum levels of cholesterol and triglycerides were investigated using an enzymatic colorimetric method (CHOD-POD; SAT-450 biochemical analyzer; Assel S.r.l., Rome, Italy).

**Figure 1:**
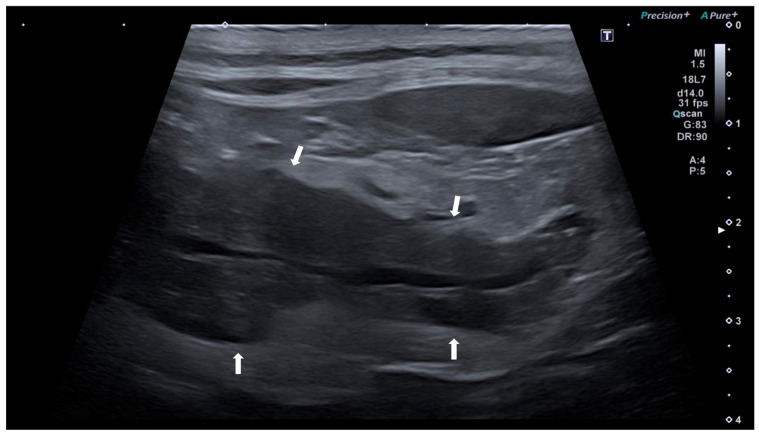
Ultrasound image of the right pancreatic limb of a dog with acute pancreatitis. The pancreas (between arrows) is enlarged, with irregular margins, hypoechoic, with homogeneous echotexture, and peripancreatic peritoneal hyperechogenicity.

**Figure 2:**
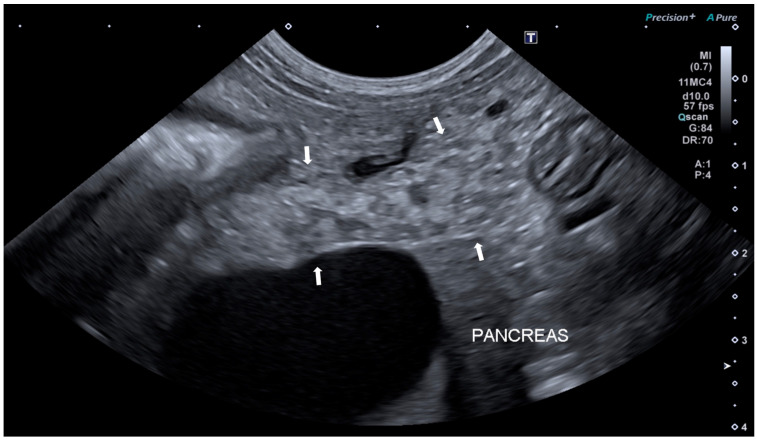
Ultrasound image of the left pancreatic limb of a dog with chronic pancreatitis. The pancreas (between arrows) is normal in size, with irregular margins, hyperechoic, and with inhomogeneous echotexture.

Based on ultrasonographic features, pancreatic ultrasound alterations were classified as acute, if an enlarged and hypoechoic pancreas with peripancreatic peritoneal hyperechogenicity and/or peripancreatic free fluid was present; or chronic, if a hyperechoic pancreas within a normal/reduced range for size, pancreatic inhomogeneous parenchyma with/without hyperechoic non-shadowing foci or lines were present [26]. Examples of acute and chronic US findings are represented in Figure 1 and Figure 2.

Hyperlipemia was defined as the presence of hypercholesterolemia (CHOL > 280 mg/dL) and/or hypertriglyceridemia (TRI > 90 mg/dL), and was subsequentially graded into mild if CHOL > 280 mg/dL or TRI > 90 mg/dL, and moderate-to-severe if both CHOL and TRI concentrations were increased.

Based on clinical history and clinical presentation, hematobiochemical profile and ultrasound, dogs were divided into two groups: cholestatic dogs with pancreopathy (PBD group = pancreatic and biliary disease) and dogs with biliary tree disease in the absence of pancreatic alterations (BD group). Dogs with concurrent pancreopathy might have either subclinical or clinical pancreopathy. Dogs were assigned to the group “pancreatic injury” if pancreopathy was subclinical, showing only ultrasonographic pancreatic alterations or a DGGR lipase increase (>143 U/L). When clinical signs (vomiting, diarrhea, abdominal pain and depression), a DGGR lipase increase, and ultrasonographic abnormalities were present, dogs were assigned to the pancreatitis group.

Non-normally distributed data, such as age and DGGR lipase, are expressed as median and range, whereas presence and type (acute/chronic) of pancreatic injury and pancreatitis are reported as categorical variables (*n*; percentage). US score and hyperlipemia, including its grading, were compared between BPD and BD dogs using a Chi-square test or the Fisher exact test depending on data distribution. Results were considered statistically significant for *p* values < 0.05.

## 3. Results

The study population (*n* = 81) was primarily composed of mix-breed (*n* = 19), followed by Labrador Retriever (*n* = 7), German Shepherd (*n* = 5), Jack Russel Terrier (*n* = 4), Pincher (*n* = 4), Border Collie (*n* = 3), Cocker Spaniel (*n* = 3), Dachshund (*n* = 3), French Bulldog (*n* = 2), Maltese (*n* = 2), English Setter (*n* = 2), Golden Retriever (*n* = 2), Cane Corso (*n* = 2), Chihuahua (*n* = 2), Bernese Mountain dog (*n* = 2), and a single dog for each of the following breeds: Alaskan Malamute, Australian Shepherd, Beagle, Belgian Shepherd, Boston Terrier, Cavalier King Charles Spaniel, Collie, Dobermann, Flat Coated Retriever, Fox Terrier, Greater Swiss Mountain dog, Maremma Sheep dog, Rhodesian Ridgeback, Shih Tzu, Schnauzer, Springer Spaniel, Welsh Terrier, West Highland White Terrier, and Yorkshire. There were 37/81 males (46%) and 44/81 females (54%) with a median age of 9.3 years (range 1–16 years). Raw data about the study population are reported in Supplementary Table S1.

**Figure 3:**
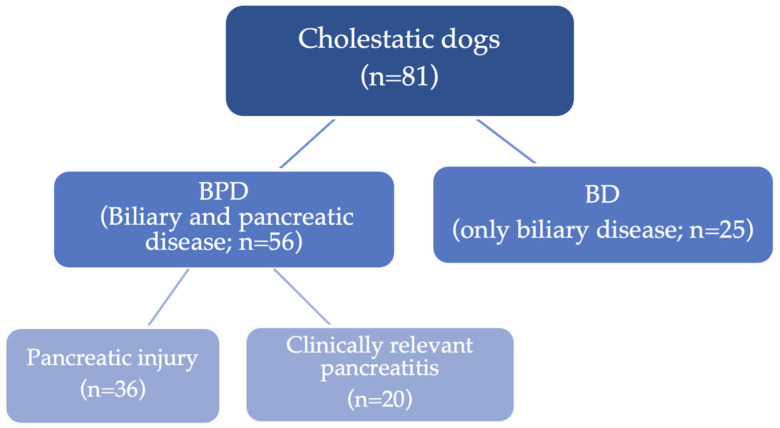
Graphical representation of the study population and its subgroups.

The population was divided into two main groups based on the previous criteria: the PBD (56/81; 69%) and BD group (25/81; 31%). Of the PBD dogs, some had “pancreatic injury” (36/56; 64%), while the others had clinically relevant pancreatitis (20/56; 36%) (Figure 3).

**Figure 4:**
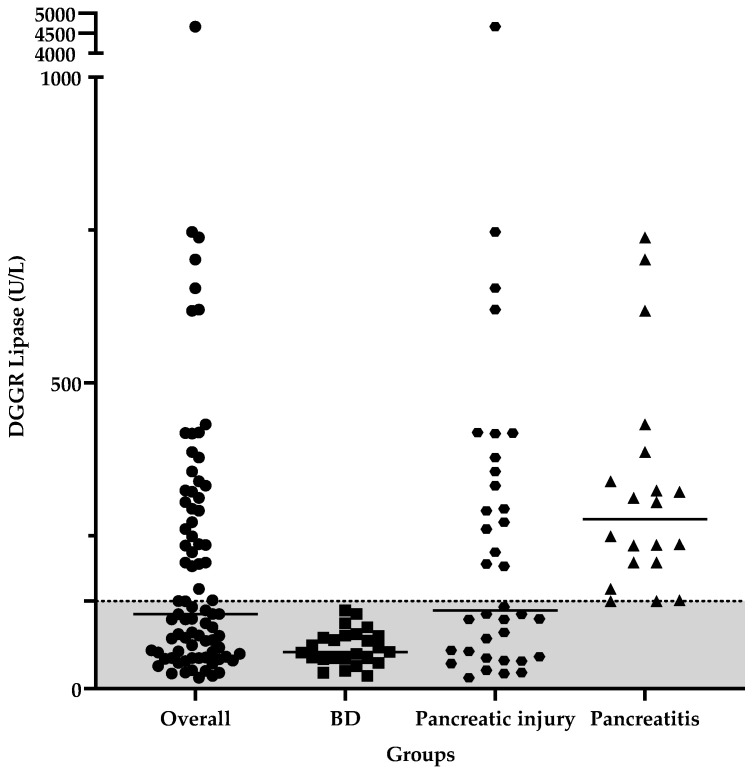
Scatterplot graph of DGGR lipase in the overall population and of the study population groups (BD and PBD [pancreatic injury and pancreatitis]). The gray band represents the normal range (<143 U/L) and the black lines in each scatterplot represent the median value.

Median DGGR lipase in the overall population was 122 U/L (range 18–4668 U/L) and its distribution is reported in Figure 4, along with the DGGR lipase of the study groups (BD group: 60 U/L, range 20.7–128 U/L; Pancreatic injury group: 127.5 U/L, range 17.8–4668 U/L; Pancreatitis group: 277 U/L, range 143–738 U/L).

Based on pancreatic ultrasound findings, 42/81 dogs (52%) showed no abnormalities, whereas in the PBD group, 24/56 dogs (43%) had chronic pancreatitis features, and 15/56 dogs (27%) had signs of acute pancreatitis. Median DGGR lipase was 113 U/L (range 30–738 U/L) and 185 U/L (range 18–702) in acute and chronic pancreatic US, respectively.

The US score of the biliary tract disease in the overall population was mild in 64/81 dogs (79%) and moderate in 17/81 dogs (21%); no dogs in this study registered a severe score. In the PBD group, 42/56 dogs (75%) had a mild US biliary tract disease score while 14/56 (25%) had a moderate score assigned. An association between US score (mild or moderate) and evidence of pancreopathy was not found (*p* = 0.18).

Sixty-six dogs (66/81, 82%) had serum hyperlipemia and its grading was mild in 27/81 dogs (34%) and moderate-to-severe in 39/81 dogs (48%). In the PBD group, 22/56 dogs (39%) had mild hyperlipemia, whereas 25/56 dogs (45%) had moderate serum hyperlipemia, while in 9/56 dogs (16%), serum hyperlipemia was absent. No association between serum hyperlipemia and its grading (mild, moderate-to-severe) and pancreopathy was found (*p* = 0.4 and *p* = 0.13, respectively).

Frequency (*n*; %) in BD and PBD groups according to the US biliary tract disease gravity score, pancreatic ultrasound features, and grade of serum lipemia are recapped in Table 2.

## 4. Discussion

Concurrent pancreopathy was found in almost 70% of the study population of dogs with biliary diseases; specifically, subclinical pancreatic injury was present in approximatively 44% of dogs, and pancreatitis was present in approximatively 24% of cholestatic dogs. Even though the relation between biliary tree disease and pancreatic injury is not fully known, our data may suggest an interconnection. This evidence may reflect the presence of pancreatic injury related to an initial inflammatory phase, which can be undetected in an abdominal ultrasound evaluation at this early stage [27]. In fact, based on the critical threshold theory of AP, the acinar pancreatic cell has protective mechanisms for the intracellular activation of zymogens [6]. Thus, only a persistent acinar damage overcoming these protective mechanisms may interrupt the clinical damage of pancreas tissue (stressor event) [6]. It is reasonable that if the stressor event is not properly controlled by the organism, and/or the patient is not adequately monitored and supported, protective mechanisms may fail, leading to surpassing the critical threshold, and causing clinical AP [6].

In our study, only 20 dogs had clinical pancreatitis, on the basis of DGGR lipase, ultrasound alterations, and compatible clinical signs. The prevalence of subclinical pancreatic injury agrees with another retrospective study, in which the authors highlighted that elevated cPLI was found in patients with inflammatory biliary disease, despite only 50% of dogs having clinical signs compatible with pancreatitis [10].

Regarding ultrasonographic features, more than half of the dogs present in this study showed no pancreatic abnormalities. However, it is important to highlight that the 43% of dogs with pancreopathy had chronic pancreatic ultrasonographic features, possibly connected to a biliary chronic inflammation. Even though no clinical signs were detected, constant pancreatic injury along with chronic biliary tree disease might lead to chronic pancreatitis, which was the most common ultrasonographic pattern detected in our PBD population.

The lack of association between US score and the evidence of pancreopathy may suggest that the ultrasonographic severity of CBTD was not directly connected to pancreatic injury. In fact, it is well-known that the occurrence of pancreatic injury is multifactorial, and there are several risk factors beyond biliary tree diseases, including genetics, environmental factors, dietary factors, drugs or toxins, endocrinopathies, and others [6,7,8].

No statistical association was found between hyperlipidemia and its grading, and the presence of pancreopathy. These data may be explained by the high prevalence of hyperlipidemia (66/81; 82%) in our population that might affect the statistical power. Furthermore, Xenoulis et al. comparing cPLI concentrations between Miniature Schnauzers with normal and increased serum hypertriglyceridemia pointed out that even though cPLI concentrations were higher in dogs with hypertriglyceridemia, a mild-to-moderate increase in triglyceride concentrations (i.e., <800 mg/dL) may not be a risk factor for a serum cPLI concentration suggestive of pancreatitis [11]. The author suggested that, in agreement with human medicine, only severe hypertriglyceridemia may be considered a cause of pancreatitis, while mild-to-moderate forms are usually a consequence of AP [11]. It is relevant to consider that cholestatic disorders and serum hyperlipemia were strictly linked, and that this abnormality was very common in the study population, despite the presence of pancreatic injury. In our study, no severe hypertriglyceridemia (>800 mg/dL) was registered, and the maximum serum triglyceride concentration was 392 mg/dL. Despite that, in the veterinary literature, primary and secondary hyperlipemia are known to be risk factors for pancreatitis, as reported by a recent study by Xenoulis et al. In that study, dogs with AP had a relevant different lipidic profile, while triglycerides concentrations were normal [12]. Specifically, dogs with AP showed higher low-density lipoprotein, lower triglyceride-rich lipoprotein, and lower high-density lipoprotein than healthy dogs [12].

This retrospective study has several limitations. Primarily, although DGGR lipase (cut-off 143 U/L) has been proven to be an effective alternative to cPLI, some authors reported that the DGGR substrate is hydrolyzed not only from pancreatic lipases, but also by extra-pancreatic lipases, thus reducing the specificity of the test [28]. On the other hand, a recent study indicated 91.7% sensitivity and 95.3% specificity of DGGR lipase for a cPLI > 200 μg/L, with a cut-off of 143 U/L [29]. Also, although the various degrees of fibrosis, nodular hyperplasia and chronic pancreatitis may share similar US appearance; it is also true that they may overlap in the clinical scenario of chronic pancreopathy. No data regarding whether biliary tree disease was a primary or secondary condition were collected; this, along with information about body condition score (BCS) and type of current diet might be an interesting record to consider in CBTD patients.

CBTD ultrasound score is a novel tool, but it requires further studies in order to validate its diagnostic and clinical utility. Finally, the lack of monitoring of pancreatic injury in relation to cholestatic disease management is due to the retrospective nature of this study. Prospective investigations on patients’ follow-ups would be desirable.

## 5. Conclusions

Subclinical pancreatic injury is more frequent than clinical pancreatitis in dogs with chronic biliary tree diseases, and it is often associated with chronic ultrasonographic pancreatic features. Based on our data, although no statistical association was found between pancreatic injury and ultrasonographic cholestatic abnormalities or hyperlipemia, pancreatic injury should be investigated in CBTD patients, due to its high prevalence, clinical significance, and multifactorial etiopathogenesis. Further studies are needed to evaluate the outcome of patients with subclinical pancreatic injury in relation to the severity of cholestatic disease. As a future perspective, the link between pancreatic injury and the prognosis of cholestatic disease should be investigated, together with the role of a therapeutic approach to pancreatic injury in the management of cholestatic dogs.

## Figures and Tables

**Table 1:** Ultrasonographic biliary abnormalities evaluated in CBTD population.

Score Type	Ultrasound Abnormalities	Points					
		0	1	2	3	4	5
Gallbladder	Biliary sludge ^a^	Absent	Grade 1	Grade 2	Grade 3	Grade 4	Mucocele
	Mobility of the biliary sludge	\	Gravity-dependent	Non-gravity dependent	\	\	\
	Cholelithiasis	No	\	Yes	\	\	\
	Gallbladder wall thickness	<2 mm	>2 mm	Cysts/mineralizations/polypoid lesions	\	\	\
Intrahepatic biliary tree and CBTD	Intrahepatic biliary tree dilatation	No	\	Yes	\	\	\
	Mineralization of the intrahepatic biliary tree	No	Yes	\	\	\	\
	CBD dilatation	No	Yes	\	\	\	\

^a^ Grade 1: biliary sludge occupies < 25% of the gallbladder’s area; Grade 2: biliary sludge occupies between 26 and 50% of the gallbladder’s area; Grade 3: biliary sludge occupies between 51 and 75% of the gallbladder’s area; Grade 4: biliary sludge occupies > 50% of the gallbladder’s area; Grade 5: for gallbladder mucocele [20].

**Table 2:** Frequency statistics in BD (only biliary disease) and PBD (pancreatic and biliary disease) groups according to US biliary tract disease gravity score (mild/moderate), pancreatic ultrasound features (normal/acute/chronic), and grade of serum lipemia (mild/moderate-to-severe/absent) expressed as number of cases and percentage (%).

Features		BD (*n* = 25)	PBD (*n* = 56)	
			Pancreatic Injury (*n* = 36)	Pancreatitis (*n* = 20)
US biliary tract disease	Mild	22 (88%)	27 (75%)	15 (75%)
	Moderate	3 (12%)	9 (25%)	5 (25%)
Pancreatic Ultrasound	Normal	25 (100%)	17 (47%)	/
	Acute	/	10 (28%)	5 (25%)
	Chronic	/	9 (25%)	15 (75%)
Hyperlipemia	Absent	6 (24%)	4 (11%)	7 (35%)
	Mild	5 (20%)	14 (39%)	8 (40%)
	Moderate-to-severe	14 (56%)	18 (50%)	5 (25%)

## Data Availability

The complete dataset is available as Supplementary Table S1.

## Supplementary Materials

The following supporting information can be downloaded at: https://www.mdpi.com/article/10.3390/ani14050795/s1, Table S1: Raw data of signalment and biochemical parameters of the study population. Reference intervals of the biochemical parameters are reported in square brackets.
