# Supplementary material for: An Association between Pancreatic and Cholestatic Biliary Disorders in Dogs
Source: Animals (Basel). 2024 Mar 4;14(5):795. doi: 10.3390/ani14050795 (PMC10931125; doi:10.3390/ani14050795)
Supplement: Supplementary file 1 [file animals-14-00795-s001.zip › animals-2832658-supplementary.pdf]

Supplementary Table S1. Raw data of signalment and biochemical parameters of the study population. Reference intervals of the biochemical parameters are reported in square brackets.

Subject	Sex	Age	Breed	Total bilirubin [0-0.3 mg/dL]	Cholesterol [128-280 mg/dL]	Triglycerides [25-90 mg/dL]	ALP [45-250 U/L]	GGT [2-11 U/L]	ALT [20-70 U/L]	DGGR lipase [<143U/L]
1	F	11.4	Welsh Terrier	44.9	777.0	347	13570	157.0	287.3	18
2	M	0.4	German Shepherd	0.1	341.9	92	355	0.3	44.5	21
3	F	11.1	Mixed breed	1.7	385.5	164	865	3.8	22.8	25
4	F	5.9	Fox Terrier	0.5	481.8	90	134	4.3	41.2	26
5	MC	10.9	Boston Terrier	0.1	453.9	89	270	15.2	106.9	26
6	F	0.3	Border Collie	0.3	378.5	95	326	2.7	11.0	29
7	MC	11.1	Jack Russel Terrier	0.4	544.4	147	439	10.3	58.3	30
8	M	2.8	Mixed breed	0.7	307.0	56	746	20.9	88.4	37
9	M	10.9	Labrador Retriever	2.5	314.4	392	558	1.0	84.8	41
10	M	1.7	Collie	0.3	241.8	50	277	1.1	48.3	42
11	M	9.4	Schnauzer	0.3	213.4	124	2551	388.0	200.6	45
12	F	9.3	Labrador Retriever	6.4	370.8	117	3059	89.6	1233.4	46
13	M	5.7	Border Collie	0.5	310.7	118	4575	21.0	93.0	48
14	M	1.1	Golden Retriever	0.2	446.5	53	265	0.8	100.1	49
15	M	12.7	Jack Russel Terrier	0.2	379.4	65	3814	509.0	2316.5	50
16	M	6.8	Belgian Shepherd	0.3	338.9	95	109	3.0	49.4	50
17	MC	9.1	Mixed breed	3.3	619.7	108	821	36.1	1024.9	50
18	F	2.0	Labrador Retriever	0.9	309.0	190	716	1.7	1549.0	51
19	MC	15.7	Labrador Retriever	0.0	285.4	36	6283	1.1	3747.0	52
20	M	1.8	Beagle	0.3	404.1	27	583	2.0	39.6	57
21	FS	9.1	Chihuahua	0.3	316.0	187	382	3.9	69.3	59
22	M	1.2	Labrador Retriever	0.4	190.6	50	934	56.1	674.9	60
23	FS	8.2	Maltese	0.7	482.8	92	4048	3.9	60.5	61
24	M	6.3	Labrador Retriever	3.4	342.7	118	2509	39.6	220.3	63
25	FS	10.2	West Highland W. T.	0.4	269.0	69	866	10.7	324.2	68
26	M	3.2	French Bulldog	0.4	93.0	50	1937	24.3	954.0	71
27	FS	10.9	Maremma sheep dog	0.5	401.4	92	575	4.8	131.7	78
28	F	12.4	Pincher	0.3	302.9	175	280	4.3	228.2	79
29	M	10.2	Mixed breed	0.1	415.1	125	332	43.1	161.7	82
30	F	7.0	Bernese Mounting dog	0.3	278.4	114	344	7.6	68.9	83
31	FS	3.8	Mixed breed	0.3	394.0	160	122	1.4	41.2	86
32	F	5.9	Mixed breed	0.5	217.6	63	293	9.1	182.3	87
33	M	7.5	Cane corso	0.3	455.5	94	832	2.3	35.1	89
34	FS	5.7	Mixed breed	0.5	401.7	86	502	9.7	213.3	92
35	M	9.2	German Shepherd	0.3	300.6	241	863	1.7	89.9	100
36	FS	11.9	Jack Russel Terrier	0.7	310.6	122	5203	74.3	633.8	107
37	M	8.8	Mixed breed	0.6	265.9	359	747	7.0	156.5	113
38	M	9.6	Cane corso	0.5	303.0	98	2090	6.6	62.8	113

39	F	13.2	Mixed breed	0.3	163.8	62	110	10.1	193.5	114
40	M	6.4	Cocker Spaniel	0.3	378.9	92	235	0.3	40.1	122
41	MC	6.2	Maltese	0.4	168.2	212	1671	5.6	98.5	122
42	FS	6.7	German Shepherd	0.5	356.9	107	172	4.0	46.4	122
43	F	13.3	Dachshund	0.2	218.5	85	393	29.2	59.0	128
44	F	15.0	Dachshund	0.2	334.0	63	285	6.0	212.8	134
45	M	13.6	Jack Russel Terrier	0.2	401.0	45	841	8.2	229.9	143
46	FS	10.1	Mixed breed	0.4	150.0	5	2902	7.8	34.6	143
47	F	10.9	Rhodesian Ridgeback	0.3	290.5	49	343	2.2	84.5	144
48	M	9.9	Cavalier King Charles Spaniel	0.2	673.4	130	2934	77.1	399.0	163
49	FS	10.5	Alaskan Malamute	0.4	425.0	95	2111	11.2	488.0	200
50	F	10.5	Greater Swiss mountain dog	0.2	486.4	148	271	4.0	67.5	204
51	FS	10.4	Cocker Spaniel	0.3	212.7	71	1068	7.9	153.2	206
52	FS	11.1	Mixed breed	0.2	302.7	84	4223	4.4	260.5	206
53	F	10.8	German Shepherd	0.4	302.1	270	193	3.3	37.9	223
54	FS	15.0	Dachshund	0.2	424.0	47	2076	31.8	79.3	234
55	FS	8.2	Flat Coted Retriever	0.7	81.4	32	941	2.2	221.0	235
56	FS	3.7	Mixed breed	4.8	129.1	135	725	16.4	134.7	236
57	FS	16.9	Mixed breed	10.4	395.0	285	19170	161.0	2726.4	249
58	M	4.8	Dobermann	0.5	318.8	92	809	4.9	100.9	261
59	M	8.2	English Setter	0.4	234.0	60	294	6.7	116.0	272
60	M	5.7	Border Collie	0.4	242.0	50	310	6.3	96.0	291
61	MC	3.3	French Bulldog	2.7	101.0	106	741	11.5	408.6	294
62	FS	8.7	Pincher	0.3	171.8	50	390	4.3	57.4	305
63	FS	9.5	Mixed breed	0.2	412.0	52	1252	4.0	32.9	312
64	MC	10.4	Mixed breed	0.7	424.5	276	1398	7.9	105.1	322
65	FS	6.1	Australian Shepherd	1.3	183.0	106	6202	23.7	79.0	324
66	M	12.2	German Shepherd	0.4	228.1	155	959	10.3	334.7	332
67	FS	13.7	Pincher	0.3	466.7	102	7300	15.9	1843.4	339
68	F	15.8	Shih Tzu	0.4	353.3	92	304	4.3	56.9	355
69	FS	12.1	Pincher	0.4	183.4	121	2254	13.9	243.3	378
70	M	11.5	Springer Spaniel	0.3	456.6	100	520	8.5	114.1	387
71	FS	12.0	Cocker Spaniel	0.4	492.8	87	3296	27.2	508.0	417
72	FS	11.5	Mixed breed	0.7	422.7	85	493	1.8	97.3	418
73	MC	11.7	Yorkshire	0.6	249.4	145	3784	306.1	731.1	419
74	M	14.5	Golden Retriever	0.2	364.9	92	907	4.0	90.7	432
75	F	7.1	English Setter	0.2	299.1	52	407	2.7	60.4	618
76	F	6.1	Bernese Mounting dog	1.8	103.0	78	1428	2.2	1152.0	620
77	FS	7.6	Mixed breed	4.1	305.5	171	213	0.0	73.1	655
78	M	10.6	Labrador Retriever	13.6	694.8	310	7899	40.4	1258.0	702
79	M	2.5	Mixed breed	1.9	30.6	12	1070	4.0	3474.8	738
80	F	4.8	Chihuahua	0.5	277.0	117	810	2.1	50.3	747

81	FS	8.6	Mixed breed	0.7	364.0	86	740	4.3	63.0	4668
